# Research on an elderly low-vision field-aid system based on augmented reality

**DOI:** 10.3389/fcell.2026.1892801

**Published:** 2026-10-02

**Authors:** Xi Chen, Xu Jing, Tinglong Liu, Pin Ju, Yige Zhang, Raymond Liu, Wenlong Liu, Like Xie, Xiaofeng Hao

**Affiliations:** 1 Eye Hospital, China Academy of Chinese Medical Sciences, Beijing, China; 2 Graduate School, China Academy of Chinese Medical Sciences, Beijing, China; 3 Center for Information Technology, Dalian Polytechnic University, Dalian, China; 4 University of Cambridge, Cambridge, United Kingdom; 5 School of Information and Communication Engineering, Dalian University of Technology, Dalian, China

**Keywords:** augmented reality, elderly low vision, quality of life, rehabilitation medicine, visual aid

## Abstract

**Introduction:**

The global population is aging rapidly. Low vision among the elderly has become increasingly common. This condition seriously affects their quality of life. This study aims to develop a low-vision field-aid system for the elderly. The system is based on augmented reality (AR). It is designed to address visual field deficiency in this population.

**Methods:**

The proposed system integrates several key technologies. These include clinical field diagnosis assistance. Other components are AR-based field expansion, precise field detection, personalized field compensation, and intelligent environmental perception. The research encompasses system design, implementation of core technologies, experimental verification, and performance evaluation.

**Results:**

Experimental verification and performance evaluation were conducted. The results demonstrate that the system effectively expands the visual field. It also enhances environmental perception. Moreover, it improves mobility safety for elderly low-vision users. The system achieves personalized field compensation. This is based on individual field defect maps. It also shows stable performance under various environmental conditions.

**Conclusion:**

This AR-based field-aid system provides an innovative technical solution. It is designed for elderly low-vision rehabilitation. The system has the potential to significantly improve visual function. It can also enhance daily living safety.

## 1 Introduction

With the acceleration of global aging, the problem of deteriorating vision among the elderly has become increasingly serious. It is mainly caused by blinding diseases such as diabetic retinopathy, glaucoma, age-related macular degeneration and retinal vascular diseases (Yuan L. Y. et al., 2025; Gagliardi et al., 2025). This has become a major public health challenge (Christensen et al., 2009; Bourne et al., 2021).

With the accelerating global aging trend, low vision among the elderly has become a major public health challenge. According to the World Health Organization (WHO), the global population aged 60 and above is projected to reach 2 billion by 2050 (United Nations Department Department of Economic and Social Affairs Population Division, 2019). In China, the aging process is particularly pronounced. The WHO International Classification of Diseases (ICD-10) defines low vision. A person with low vision has visual acuity between 6/18 and 3/60 in the better eye. Their field of vision is between 20 and 30° (Vashist et al., 2017). This condition severely restricts daily activities. Examples include reading, writing, watching television, and using electronic devices (Cheng et al., 2023; Loomis et al., 2018; Reeves, 2011; Wood, 2022; Luo et al., 2026). According to the WHO, there are at least 2.2 billion people globally with visual impairment (Agarwal and Tripathi, 2021). This condition reduces independent living ability (Riazi et al., 2022). It also leads to low employment rates. As a result, it increases family care burden. Socioeconomic costs rise as well (Hernandez-Moreno et al., 2020).

The primary function of low vision assistive devices is to provide angular magnification, thereby enlarging the perceived size and reducing the perceived viewing distance of objects to facilitate visual recognition. Traditional low-vision aids include magnifiers and other optical devices (Minto and Butt, 2004; Ager, 1998; Jackson et al., 2017). They can enhance visual functions and are applied in diseases such as macular degeneration, diabetic retinopathy, and optic nerve atrophy (Sapkota and Kim, 2017; Flaxman et al., 2017; Dalal and Shah, 2022). However, the effects are limited and there are obvious drawbacks. For instance, magnifiers provide a narrow field of view. Most assistive devices are single-function. As a result, they cannot meet the diverse visual needs of elderly low-vision patients, especially in complex and changing environments (Kaur and Gurnani, 2023; Perrault et al., 2023). Conventional rehabilitation approaches have other problems too. They lack personalized adaptation. They also lack real-time environmental perception. Hazard warning capabilities are missing as well. Because of these limitations, they cannot properly address the visual field defects or daily safety risks faced by this population (Musa et al., 2025). Therefore, there is an urgent need to develop more advanced and intelligent low-vision rehabilitation technologies.

In recent years, AR has achieved rapid development and demonstrated broad healthcare applications (Bruno et al., 2022; van Doormaal and van Doormaal, 2024; Prunoiu et al., 2022). AR also plays a significant role in ophthalmic diagnosis and treatment (Yuan L. et al., 2025; Delikaya et al., 2026; Jia-Wen et al., 2026). Augmented reality overlays virtual information onto the real environment. This creates immersive interactive experiences. AR has already been used in surgical navigation, medical education, and rehabilitation training (Zhao et al., 2021; Edalati et al., 2024; Su et al., 2023). Introducing AR into low-vision rehabilitation could help overcome the technical limits of traditional assistive devices. It may also offer more intelligent and personalized solutions for visual assistance (van Nis et al., 2020; Cao et al., 2025).

Therefore, this study aims to design and develop an elderly low-vision field-aid system based on augmented reality. The core objective is to address visual field defects in elderly low-vision patients. To do this, the system integrates two major functional modules. One is clinical visual field diagnostic assistance. The other is AR-based visual field expansion. The system is intended to enhance visual perception ability in these patients. It also aims to improve their environmental safety. The significance of this research includes (Yuan L. Y. et al., 2025): This provides an innovative technological pathway for elderly low-vision rehabilitation. It is expected to significantly improve their quality of life (Gagliardi et al., 2025). This promotes the deep integration of AR in rehabilitation medicine. It also supports the practical application of AR in this field (Christensen et al., 2009). This offers theoretical support for related product development. It also provides technical references for policy formulation. In doing so, it actively responds to the national strategy for addressing population aging.

## 2 Methods

### 2.1 Design of vision-assistance system based on augmented reality

The hardware architecture has several parts. It includes a camera, a processing unit, and an output AR display. The processing unit handles real-time visual field analysis and AR rendering. This is shown in Figure 1. The processing unit can be built as an embedded module. Alternatively, it can be offloaded to a companion device like a smartphone. This enables flexible deployment. It also reduces wearable complexity.

**FIGURE 1:**
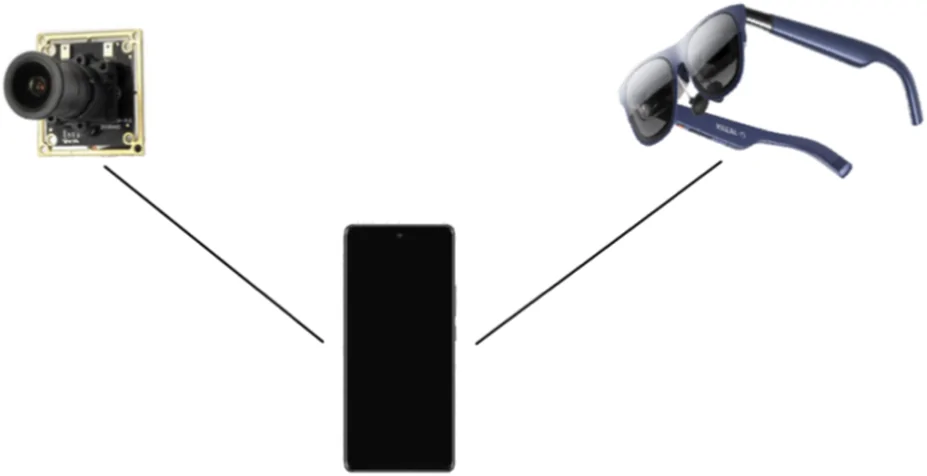
System hardware architecture.

### 2.2 Visual field and defect detection

The patient wears an AR device. They are instructed to keep looking at a central cross displayed straight ahead. One point from the dot matrix is randomly selected. This point briefly enlarges and brightens for a single flash. The flash happens at random time intervals. When the patient sees the flash, they press a hand-held button. The system records the perceived stimuli as visible points. Throughout the test, real-time voice prompts are given through the AR glasses speakers. These prompts guide the patient through the required steps.

The system uses the spatial coordinates of these visible points. It then calculates the corresponding horizontal and vertical visual angles. These angles are measured relative to the center of the patient’s eye. A set of visual angles is obtained. Finally, this set helps determine the patient’s effective visual field range. It can also show the extent of any visual field defects (blind spots).

Figure 2 presents a representative outcome of the visual field assessment. The central cross is the fixation point. The patient must keep looking at it throughout the test. The surrounding points show where visual stimuli appear. The filled dark points mark stimuli that the patient saw. The empty circles mark locations that were not detected. The pattern of detected points shows how much of the visual field is working. If there are gaps or missing areas, that means the patient has visual field defects.

**FIGURE 2:**
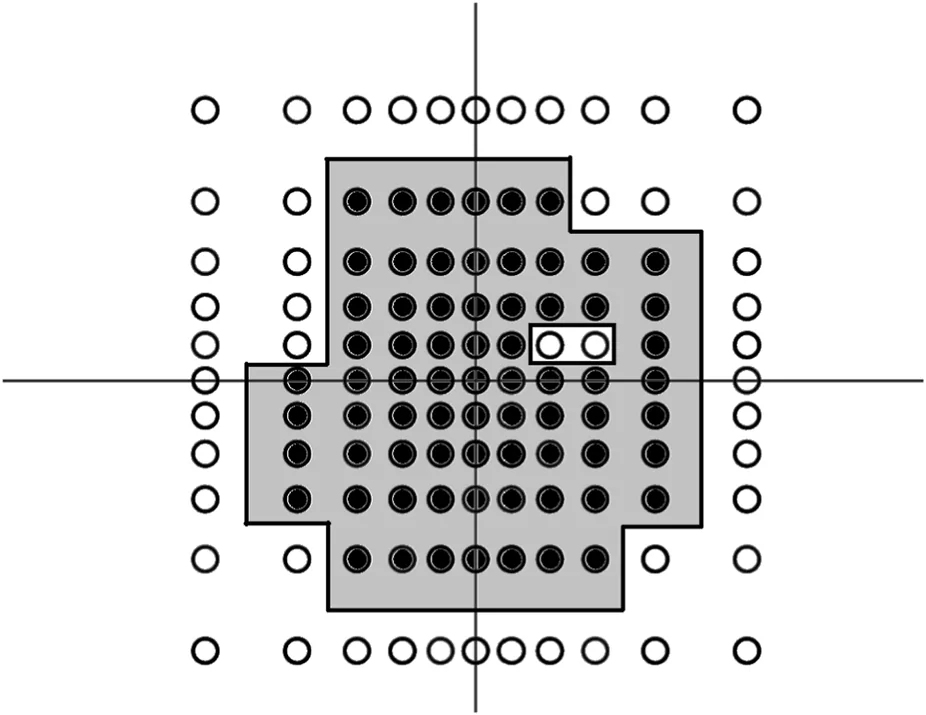
Example visual field test results.

Figure 3 illustrates the geometric framework used to compute the visual angles associated with each detected stimulus shown in Figure 1. The eye is treated as the reference origin. It fixates along a central axis toward a flat display screen. A stimulus appears at a certain position on the display. This position is described by its horizontal (X) and vertical (Y) distance from the fixation point. These spatial offsets are used to calculate the corresponding visual angles α and β. These angles measure how far the stimulus is from the line of sight. The formulas are α = arctan (X/D) and β = arctan (Y/D). Through this conversion, we turn spatial positions into angular coordinates. The detected points shown in Figure 1 can then be mapped into a visual field representation. This allows a quantitative assessment of the patient’s visual field extent. It also helps identify potential defects.

**FIGURE 3:**
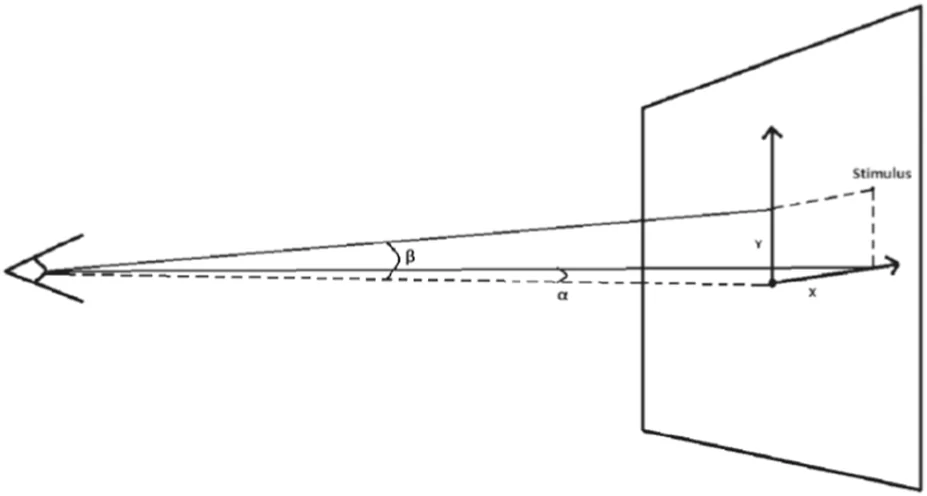
Visual angle mapping.

### 2.3 Display of normal visual field

First, the camera captures a video that shows a normal visual field range. Then, based on the patient’s measured visual field, the video is scaled proportionally. After scaling, it is presented inside the patient’s actual visual field. By adjusting the size and position of the video in this way, the approach avoids blocking too much of the patient’s remaining vision. At the same time, it still effectively shows what a full normal visual field looks like.

Figure 4 shows the concept of AR-based visual field compensation. The image on the right shows the full scene captured by the camera. The image on the left simulates the patient’s restricted visual field. In this simulation, only a limited central region is visible. The surrounding area is hidden. To help the patient, the system extracts useful visual information from outside the visible area. This information is then processed and overlaid inside the patient’s remaining functional field of view. This is shown in the inset image. In this way, the user can perceive previously unseen areas through a compressed or repositioned view. This improves situational awareness. It also reduces the impact of visual field loss.

**FIGURE 4:**
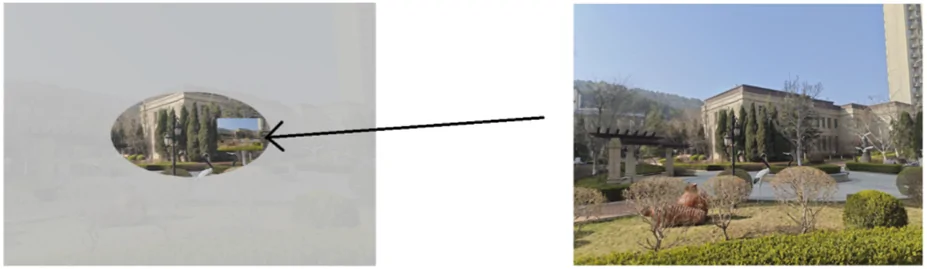
AR-assisted visual field compensation.

## 3 Results

### 3.1 Implementation and results of visual field detection

We start with human visual field angles. We also consider how visual perception is distributed across the field. Then, using spatial geometry, we generate a dot-matrix cloud. This cloud has multiple quadrants. It also has high density and uniform point distribution. This design ensures effective coverage of the visual field. It also improves the accuracy of localizing visual defects. The visual angle range is central 30° (field of view ±30°), with an angular resolution of 5° (angular interval between test points). The system uses a lightweight mobile architecture. It runs on the Android operating system as the application environment. AR glasses are used for visual presentation. Together, they create a collaborative mode where the smartphone controls the process and the AR glasses show the display. This is illustrated in Figure 5. This design overcomes the spatial limits of traditional perimeters. It also supports flexible use in different settings. These include outpatient clinics, hospital wards, and physical examination centers.

**FIGURE 5:**
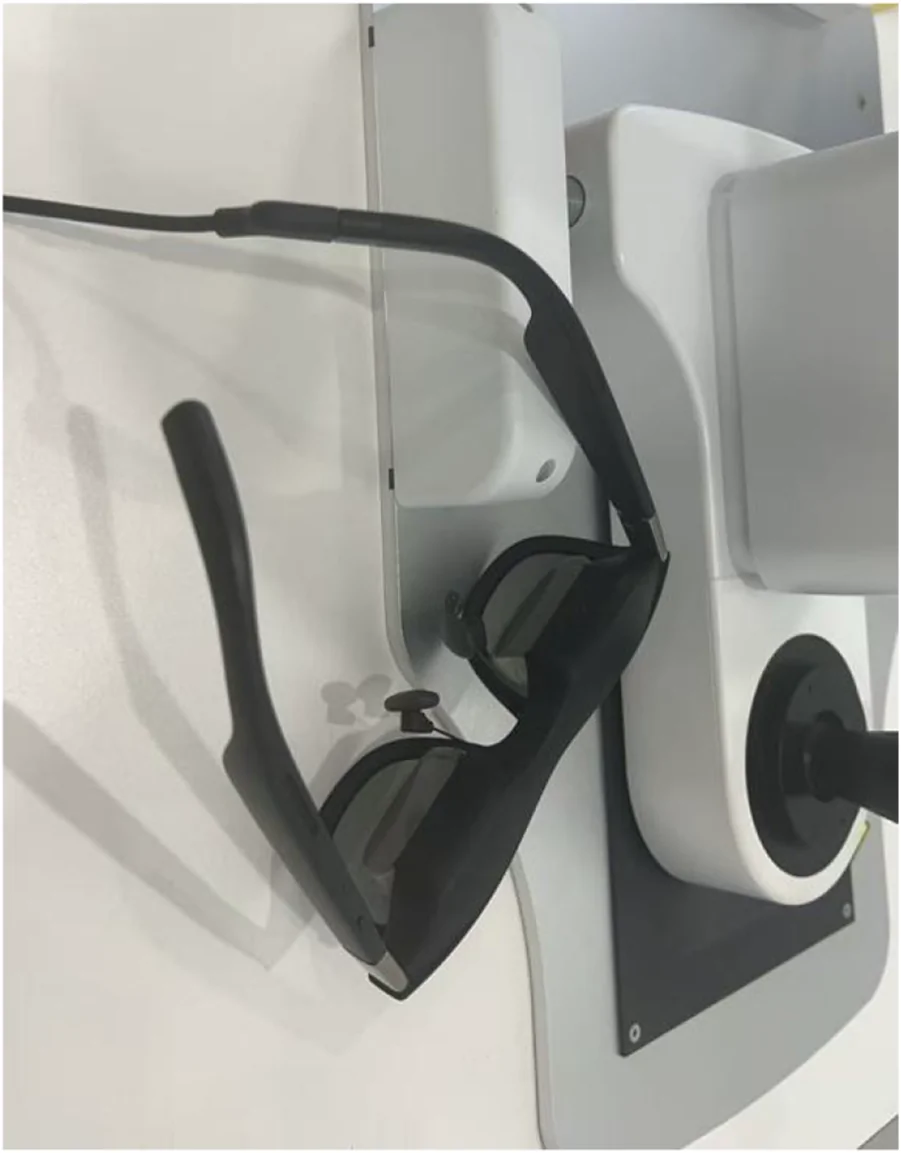
AR hardware device.

The software uses low-latency data transmission. It also uses real-time response technologies. This achieves millisecond-level synchronization between light stimulus triggering and user marking. As a result, the examination data are authentic and reliable. The system is built with modular programming and a layered architecture. This improves stability and compatibility. It can adapt to different models of Android smartphones and AR glasses. It also reduces hardware integration costs. The system provides data statistics and quadrant analysis. It automatically classifies, calculates, and visualizes examination data. Visual field defects are shown through graphical displays. The system also uses secure data encryption and local storage. This protects patient privacy and ensures data security.

The user interface follows simple and easy-to-use principles. It is designed for medical software. The operational logic matches the working habits of healthcare professionals. Users can become proficient quickly without extensive training. Overall, this technical solution balances professionalism, portability, and practicality. It does so by combining advanced AR technology with clinical ophthalmic examination standards. This enables visual field testing that is intelligent, convenient, and precise. As a result, it effectively improves the overall standard of ophthalmic visual field examination.

The system provides an information entry page for recording the subject’s name, gender, age, and email address. The entered information can also be stored within a drop-down control for convenient selection and management. The system also displays simplified examination results in the form of coordinate-based graphical plots.

### 3.2 Data processing and visual field analysis

Development of a Visual Field Data Acquisition and Analysis System.

A data acquisition system was developed. It connects to visual field examination devices. The system collects patient examination report data in real time. It acquires visual field examination results. Then it stores the data on a server. After that, the system analyzes the reports. This analysis accurately determines the patient’s visual field range. It also performs specialized analysis. The subject follows on-screen instructions to carry out the visual field examination. This is shown in Figure 6. The system records response information at the same time. This information is used for statistical analysis. It is also used later for graphical visualization of the examination data.

**FIGURE 6:**
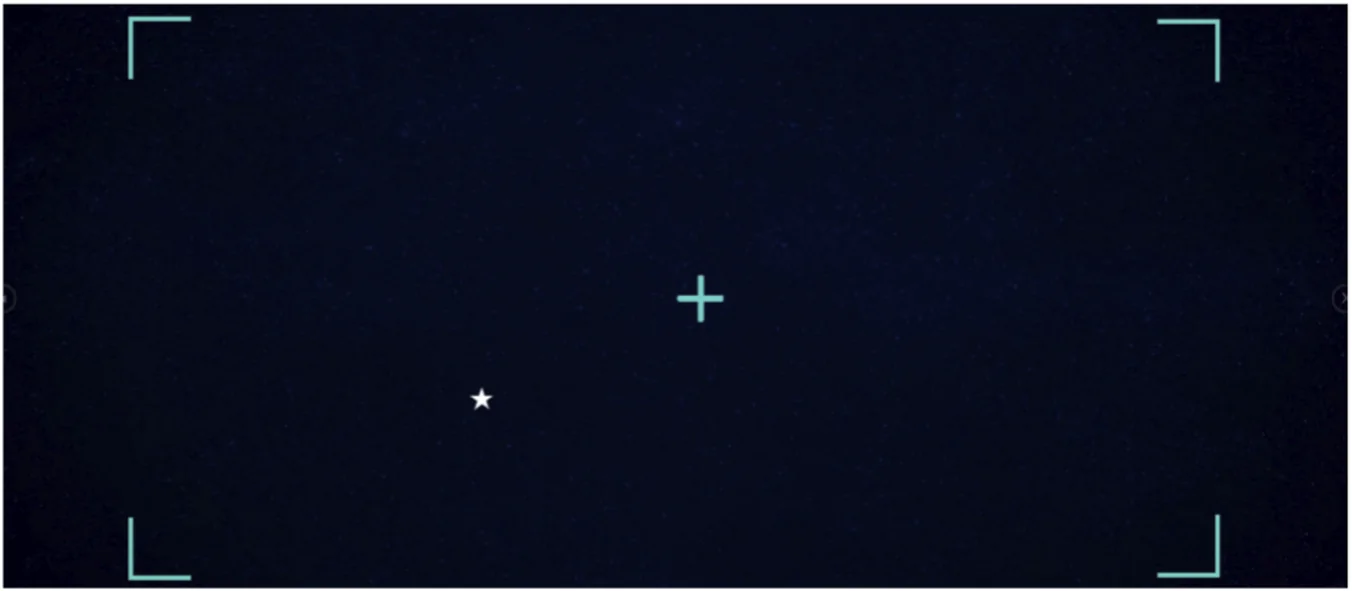
Visual field examination procedure.

The available examination modes include left eye, right eye and both eyes. During left-eye testing, the subject must cover the right eye and look straight ahead with the left eye. When a flashing star marker is observed, the response button is pressed. During right-eye testing, the subject must cover the left eye and look straight ahead with the right eye. When the subject sees a flashing star marker, they press the response button. During binocular testing, the subject looks straight ahead with both eyes. If a flashing star marker appears, they press the button again.

Single or multiple examination items can be performed according to the testing requirements. The interface marks completed examination items. Users can then choose untested items or submit the information directly. After submission, the results become available. The interface shows all results from that submission. Untested items have empty result fields. Completed items show their results. Clicking on a result item opens a detailed view. This is illustrated in Figure 7. Results are shown as a four-quadrant matrix. Recorded points get a value of 1. This means the subject saw the point. Unrecorded points get a value of 0. These binary values (0 and 1) are used for visual field defect analysis, data processing, and assessment of visual impairment.

**FIGURE 7:**
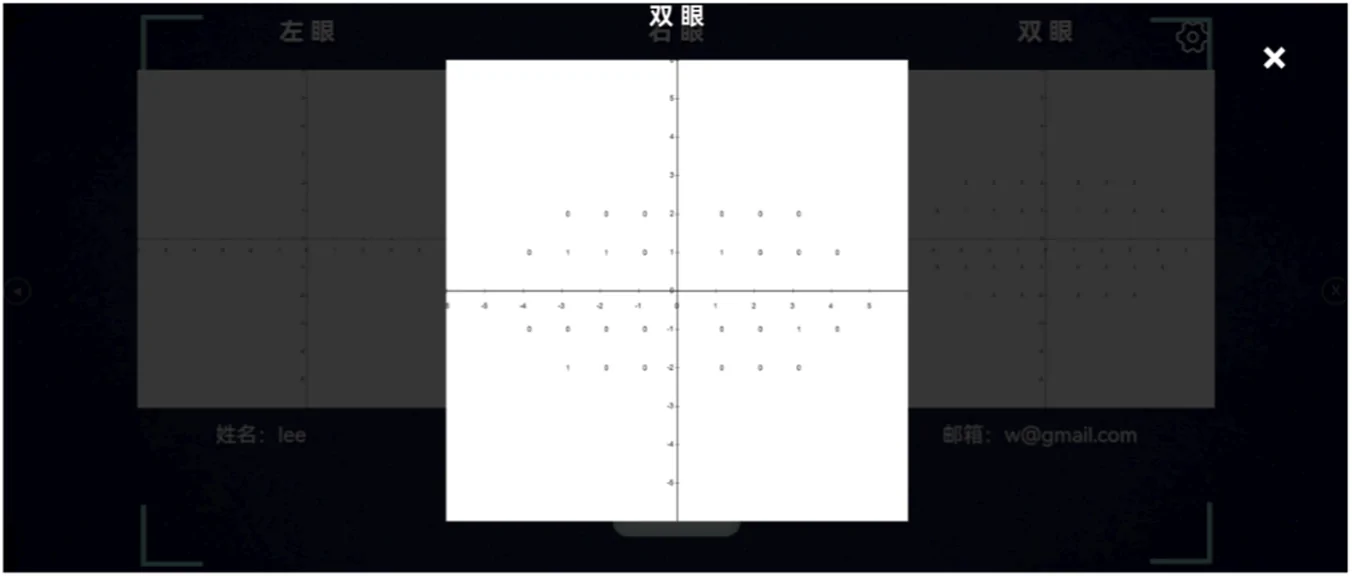
Report of visual field examination.

### 3.3 Implementation and demonstration of the visual field expansion function

This module has several main tasks. It captures external visual scenes efficiently. It also handles cross-device transmission. Finally, it performs spatially integrated display on the AR side. The system creates a transmission chain. This chain uses a “camera-smartphone-XREAL glasses” architecture. It achieves a low-latency closed loop. Through this loop, visual data from the physical world are transferred into AR space. The specific technical implementation details are as follows.

The system handles UVC-based image capture. It also manages USB hot-plugging and low-level driver adaptation. On the Android side, it deeply integrates the USB Host API with an open-source UVC driver framework. This overcomes Android system restrictions on non-standard camera permissions. As a result, the system supports plug-and-play for UVC-compliant cameras. A dynamic USB device broadcast receiver was designed. It monitors ACTION_USB_DEVICE_ATTACHED and ACTION_USB_DEVICE_DETACHED events. This enables millisecond-level response to device hot-plugging. The system can also reconnect automatically. Endpoint configuration is used for low-level camera control. This includes dynamic resolution switching, frame-rate adjustment, and exposure compensation. For high-resolution video streams, memory-mapping technology is used. Data is read directly from USB isochronous transfer endpoints. This avoids memory copy overhead in the JVM heap. As a result, CPU usage and acquisition latency are significantly reduced.

### 3.4 Cross-device link collaboration and protocol forwarding

The smartphone acts as both the computational hub and the routing node. It buffers and encapsulates raw or encoded video streams from UVC. To optimize network transmission, the system uses H.264 or H.265 hardware encoding. This provides lightweight video compression. Compression reduces the bandwidth needed during transmission. Using the official XREAL SDK, a wired communication channel is set up between the smartphone and the AR glasses. The encapsulated video stream is then converted to match the rendering input specifications of the XREAL platform. This ensures timing synchronization and low bit-error-rate transmission between the smartphone and the AR device.

Based on the visual field examination results, patients can project panoramic video content into the intact regions of their visual field arranged in a nine-grid layout. By default, the projected supplementary visual content is displayed in the upper-left area of the interface as shown in Figure 8.

**FIGURE 8:**
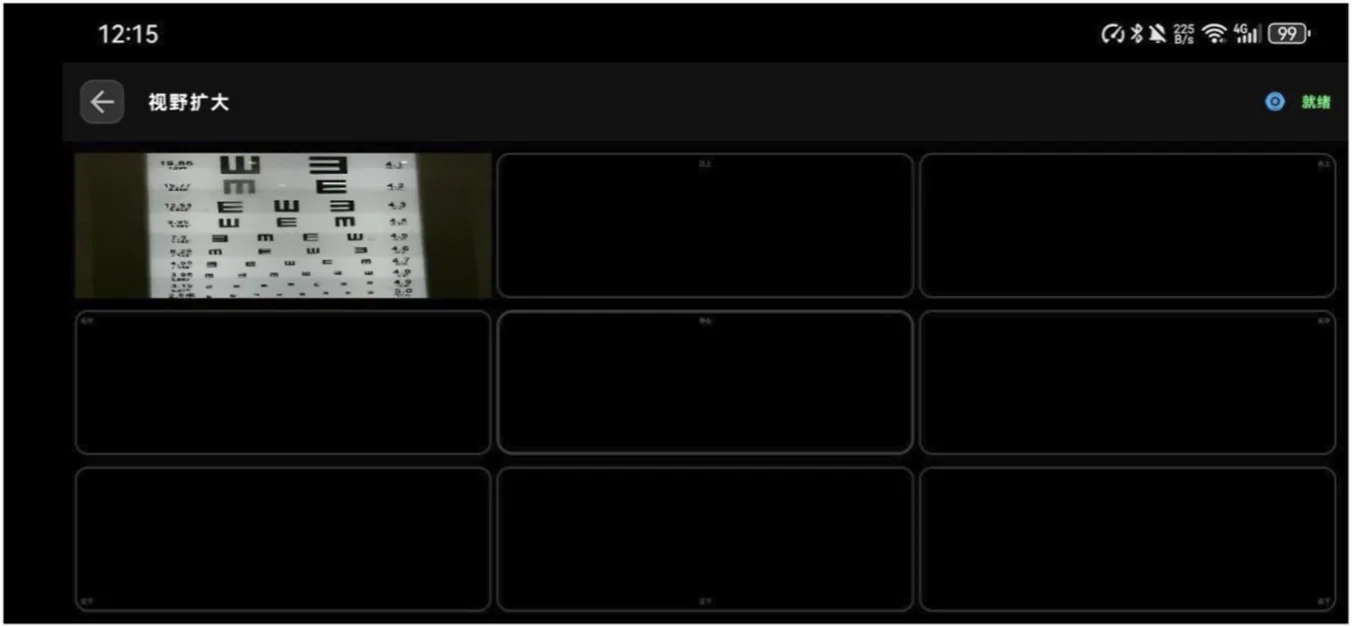
Default projection angle.

### 3.5 Experiment and analysis

This experiment was designed to achieve a quantitative evaluation of the AR system’s actual effectiveness in both visual field defect measurement and functional field expansion, ensuring the reliability and reproducibility of the experimental results.

#### 3.5.1 Simulation of visual field defects

The simulation of visual field defects was achieved by placing physical occlusions on the inner surface of the curved mirror (the combiner) of the XREAL AR glasses. This method is grounded in the optical principle that this curved mirror is the final optical interface where the virtual display image converges and enters the eye. By attaching opaque materials of specific shapes to this surface, we can precisely block light from corresponding regions of the display, thereby creating well-defined scotomata in the user’s perceived field of view. Because the occlusion is placed directly in the optical path and very close to the eye, it creates a distinct and stable defect boundary in the visual field, effectively mimicking the perceptual experience of genuine retinal or neurological visual field loss.

#### 3.5.2 Visual field defect measurement and quantitative characterization

To establish a quantified baseline of the simulated visual field defects, a customized perimetry device—the Starfield Flicker Perimeter—was employed for precise visual field measurement. Taking the right eye inferior-temporal quadrant complete defect simulation as a representative example, the experimental configuration was as follows: a physical occluder was applied to the lower-right region of the AR glasses’ combiner, and a standardized test panel was positioned at a distance of 1.5 m from the participant. The test panel contains 25 red stimulus points, evenly distributed across the four quadrants. Each quadrant has 6 points, plus 1 central fixation point. It is specifically designed to assess the integrity of the right eye’s monocular visual field. Each stimulus was presented for 300 m, with a random inter-stimulus interval of 1.5–2.5 s to prevent anticipatory responses, and the total test duration was approximately 3–5 min per participant, depending on reaction time. The response threshold was defined as the participant’s ability to press a handheld button within 1.5 s of stimulus onset; responses beyond this window were recorded as misses.

Upon wearing the test apparatus, the participant’s effective field of view exhibited a clearly demarcated scotoma in the lower-right quadrant, as documented in the visual field plot of Figure 9.

**FIGURE 9:**
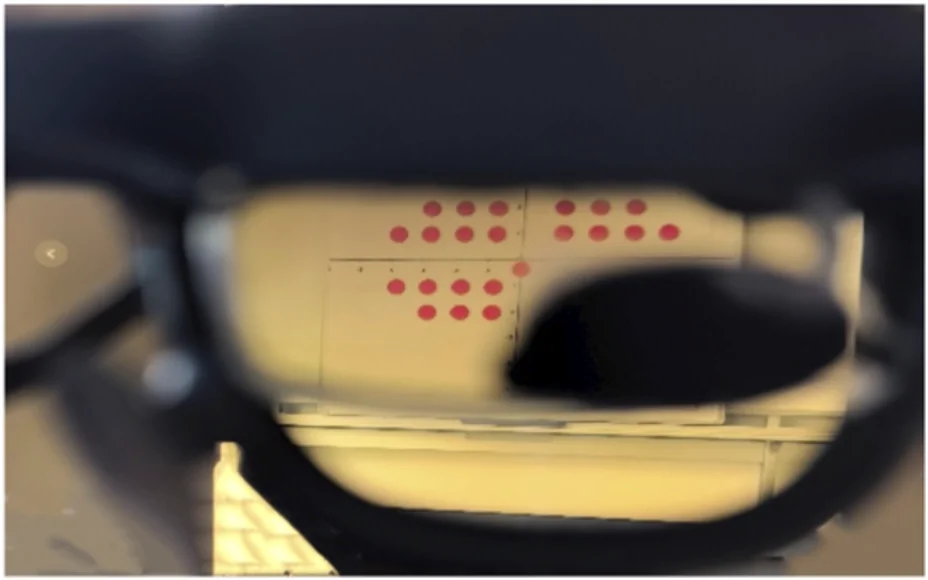
Visual field through the glasses after occlusion.

The perimetry assessment yielded high-resolution spatial mapping of the defect boundaries, enabling quantitative characterization of the induced visual field loss. The detailed breakdown of the visual field defect is presented below, following the standardized coordinate system and scoring protocol of the perimeter.

As shown in Figure 10. Visual field measurement was conducted using the standardized Starfield Flicker Perimeter, whose coordinate system defines the central fixation point as the origin (0,0). On the X-axis, negative values (−1 to −5) denote the nasal direction and positive values (1–5) denote the temporal direction; on the Y-axis, positive values (1–5) denote the superior direction and negative values (−1 to −5) denote the inferior direction. Each integer increment corresponds to a 10° visual angle, with a maximum measurement range of 50° per axis. The scoring convention assigns a value of 1 to visible stimuli and 0 to complete scotomata. Quadrant-by-quadrant analysis revealed the following: in Quadrant 1 (upper-right), only the region spanning Y = 1, 2 and X = 1, 2, 3 was visible, while areas with Y ≥ 3 and X ≥ 4 lacked valid data and were classified as defective; Quadrant 2 (upper-left) and Quadrant 3 (lower-left) exhibited intact perception at all designated test points (Y = ±1, ±2; X = −1, −2, −3), though no valid data were obtained for the more peripheral regions beyond these ranges; Quadrant 4 (lower-right) showed complete scotomata at all test points, with the entire quadrant classified as a region of absolute defect. Quantitative characterization indicated a complete 50° defect across the entire inferior hemifield, with the superior hemifield preserving vision only within a restricted rectangular zone extending 20° superiorly and 30° laterally in each direction. This defect pattern is consistent with inferior altitudinal hemianopia of the right eye, a condition typically associated with lesions of the superior occipital visual cortex and characterized by the absence of physiological binocular compensation.

**FIGURE 10:**
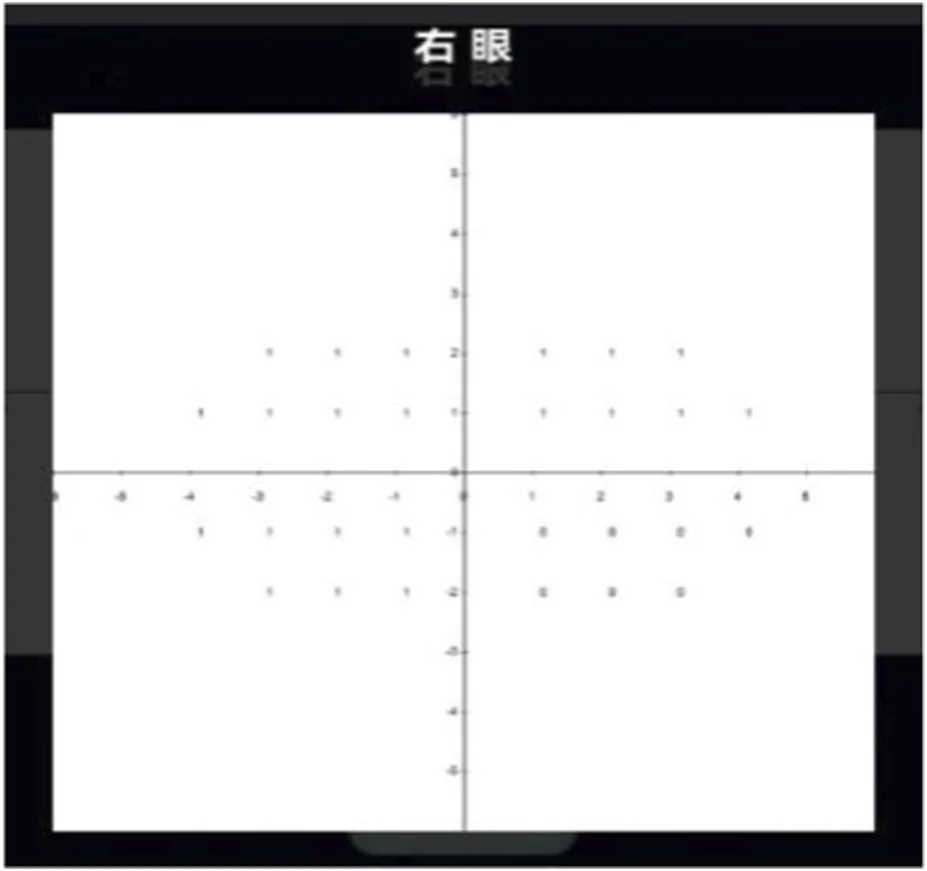
Results of the visual field expansion device test.

#### 3.5.3 Visual field expansion and compensation verification

The above results demonstrate that the perimetry device can accurately identify and quantitatively characterize the induced visual field defects. Subsequently, the visual field expansion system was connected to the AR glasses. The system first captured a video showing the normal visual field range through a camera. Based on the participant’s measured visual field, this video was then scaled proportionally and presented inside the participant’s actual visible field. As shown in Figure 11. By adjusting the size and position of the video, the approach effectively displayed complete normal visual field information while avoiding excessive occlusion of the participant’s remaining vision.

**FIGURE 11:**
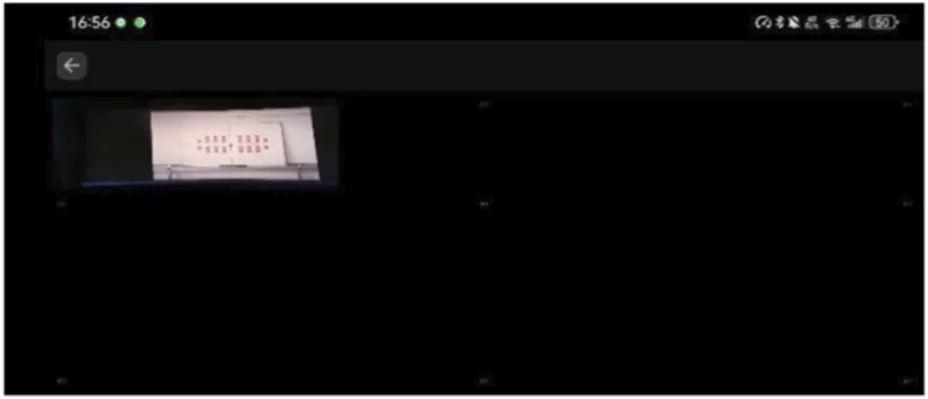
Visual field through the glasses after using the expansion device.

The post-compensation assessment revealed that the entire visual scene was clearly perceivable within the participant’s functional field of view, thereby achieving the objective of effective visual field expansion.

#### 3.5.4 Comprehensive experimental validation across defect types

Twenty participants were recruited for this experiment. The experiment employed simulated visual field defects commonly seen in clinical practice (Abraham et al., 2024; Liu et al., 2024; Uno et al., 2023; Runswick et al., 2023; Manou et al., 2022; Bjelica et al., 2021), including tubular vision, homonymous (right-sided) hemianopia, and central scotoma.

1. Tubular Vision: A circular opaque mask with a central aperture was placed on the combiner. This blocks the entire peripheral display, leaving only a small central window of light transmission, replicating the severe constriction of the peripheral visual field characteristic of tubular vision. The specific style is shown in Figure 12.
2. Homonymous (Right-Sided) Hemianopia: An opaque film was applied to completely cover the right half of the combiner’s surface for each eye. This blocks visual information from the right hemifield of the display in both eyes simultaneously, accurately simulating a right-sided homonymous hemianopia, where the same half of the visual field is lost in each eye.The specific style is shown in Figure 13.
3. Central Scotoma: A small, centered opaque dot was affixed to the combiner. This obscures the central area of the display while leaving the surrounding periphery visible. This arrangement effectively mimics a central scotoma, where central vision is impaired but peripheral vision remains intact. The specific style is shown in Figure 14.

**FIGURE 12:**
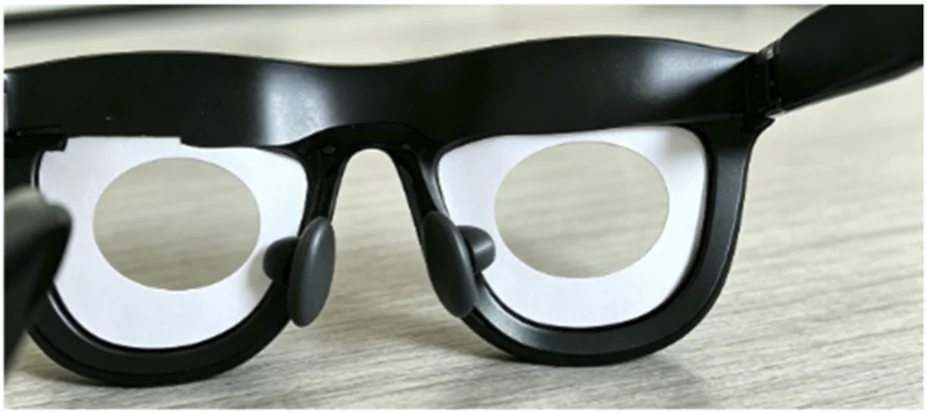
Tubular vision glasses simulation design.

**FIGURE 13:**
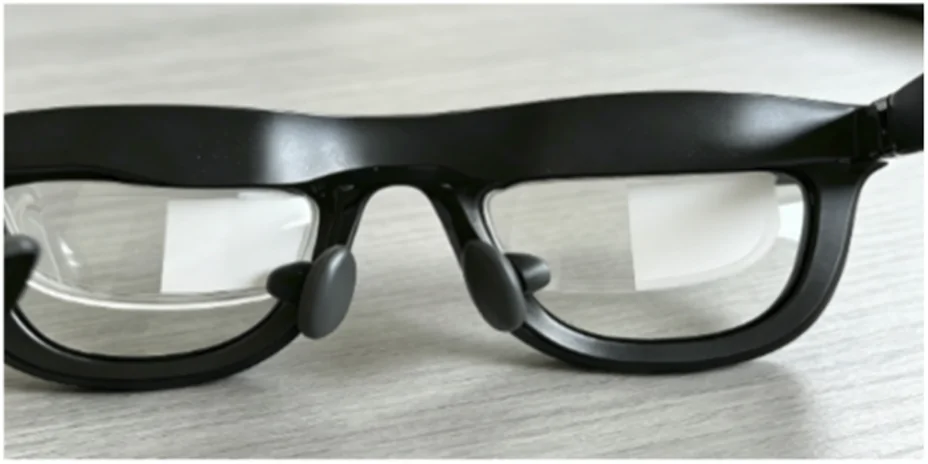
Homonymous (right-sided) hemianopia glasses simulation design.

**FIGURE 14:**
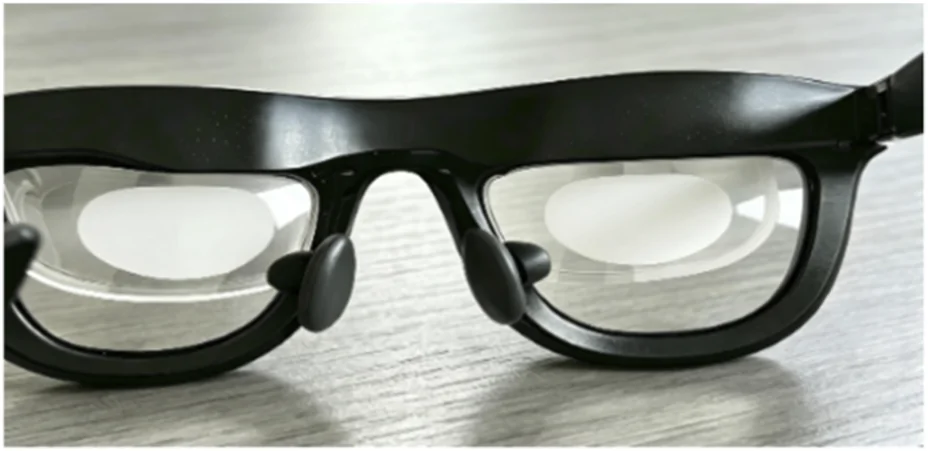
Central scotoma glasses simulation design.

By placing these precisely shaped occlusions on the curved mirror itself, the system reliably and repeatedly establishes a baseline condition of restricted visual input. This serves as a controlled “defective” state against which the compensatory projection strategy of the AR system can be compared, ensuring that the observed improvements in the visibility test are directly attributable to the remapping of information from the occluded (non-visible) areas to the participant’s remaining functional field of view.

Each participant underwent simulation tests for all three types of visual field loss. In the first phase of the experiment, all participants completed a visibility test for signage placed within a 1.5-m range while their vision was occluded. Participants were instructed to maintain fixation on a central cross displayed straight ahead to ensure stable eye position throughout the test. Stimulus points on the signage briefly brightened or flashed at random time intervals. When a participant clearly perceived a stimulus, they pressed a hand-held button to confirm detection; if the signage was not visible, no action was taken. The system recorded the spatial coordinates of all perceived stimuli and calculated the corresponding horizontal and vertical visual angles relative to the center of the participant’s eye. As shown in Figures 15–17. From this set of visual angles, a precise map of each participant’s visual field defect area was constructed, providing baseline data for subsequent personalized compensation.

**FIGURE 15:**
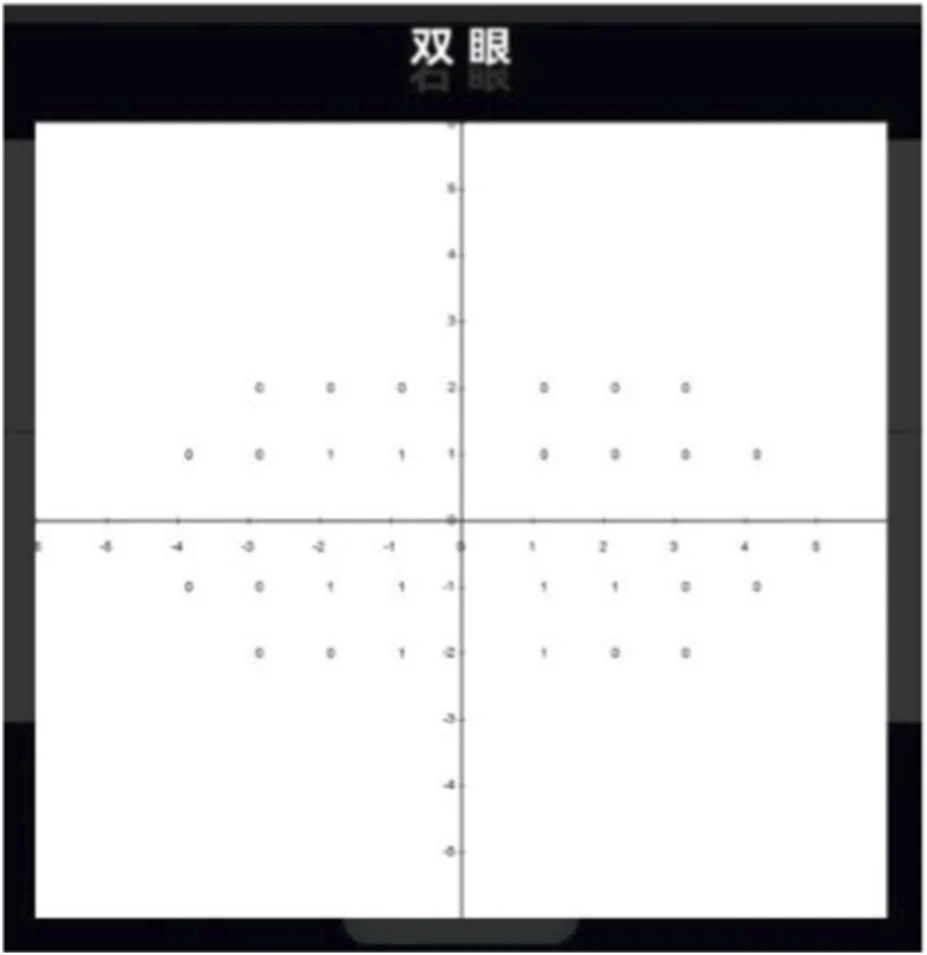
Test results of tubular vision on the visual field perimeter.

**FIGURE 16:**
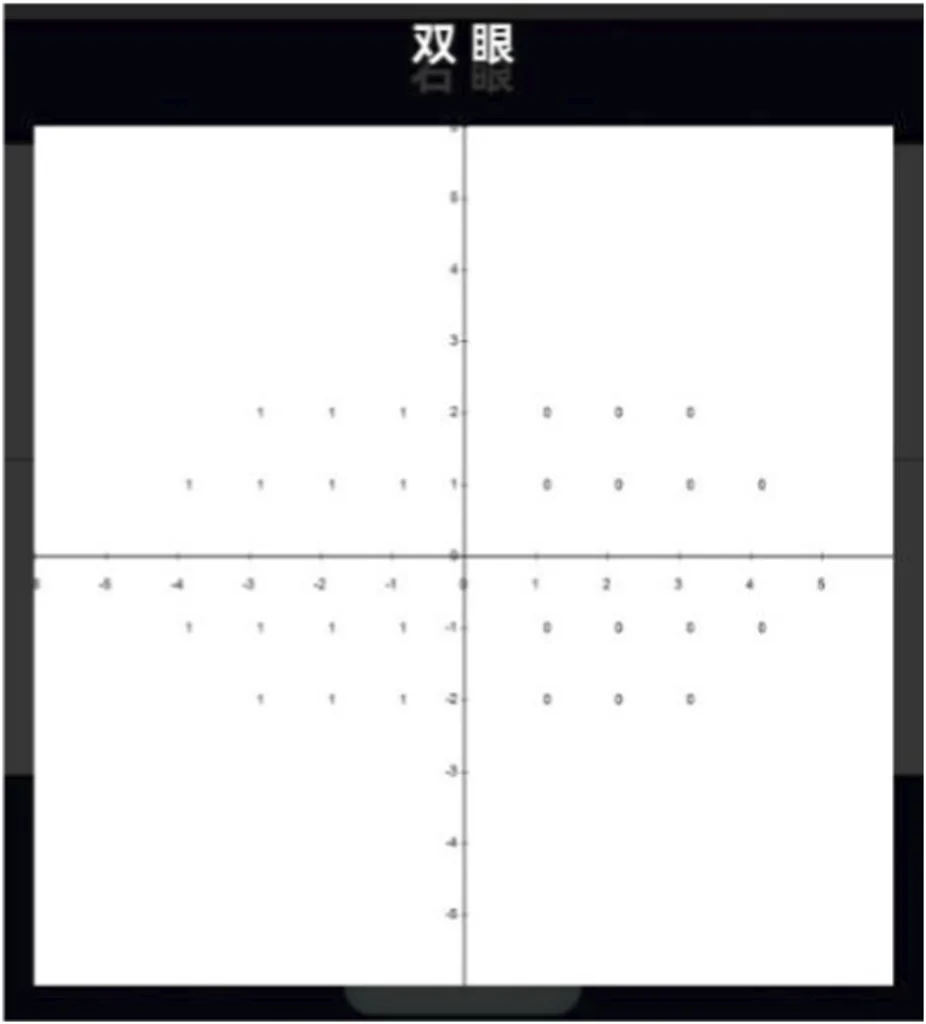
Test results of homonymous (right-sided) hemianopia vision on the visual field perimeter.

**FIGURE 17:**
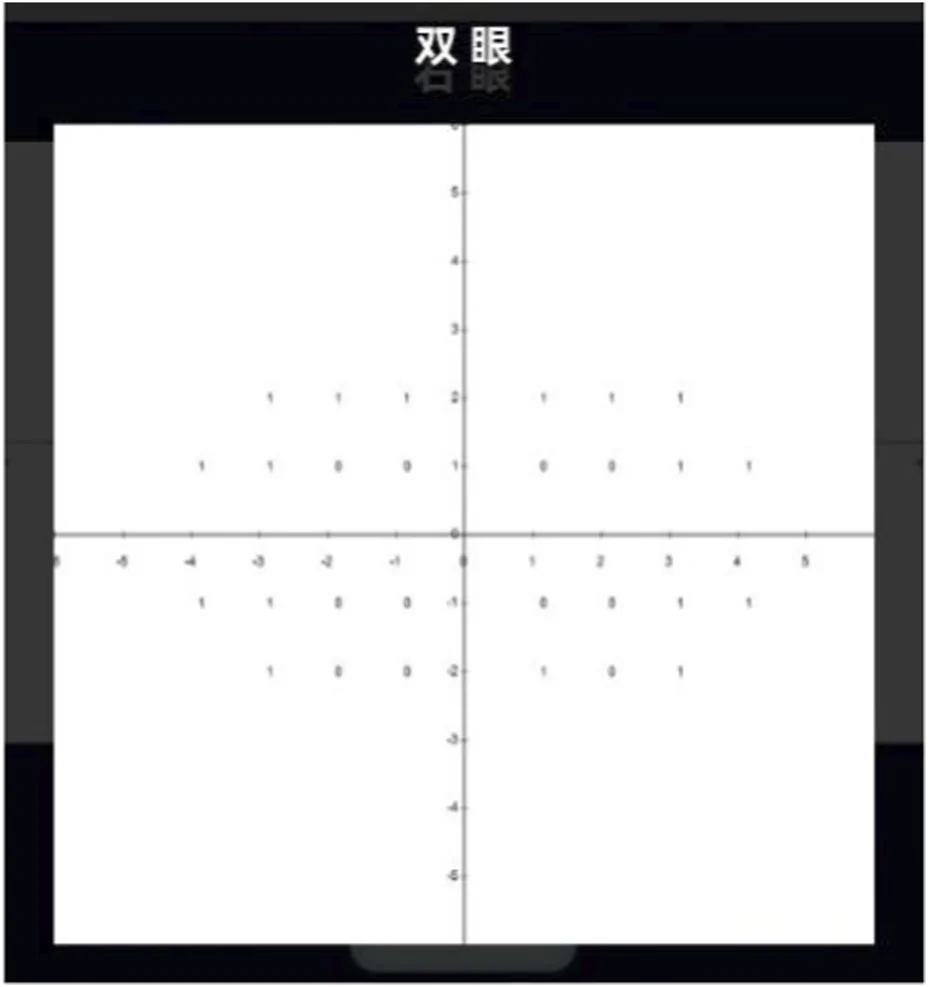
Test results of central scotoma vision on the visual field perimeter.

After completing the baseline test, participants wore precisely calibrated AR glasses connected to the intelligent visual compensation system. The same signage visibility test was then repeated, with the focus on observing whether the AR system could extract useful visual information from previously invisible peripheral areas and overlay it, in a compressed or remapped form, within the participant’s functional field of view. The experimental results are summarized in Table 1.

**TABLE 1:** Visibility test results under different simulated visual field defect conditions.

Simulated visual field defect	Experimental condition	Signage visibility
No occlusion (baseline)	Normal AR display	Visible
Tubular vision	Defective (No compensation)	Not visible
	Compensated (remapped to visible area)	Visible
Homonymous (right-sided) hemianopia	Defective (no compensation)	Not visible
	Compensated (remapped to visible area)	Visible
Central scotoma	Defective (no compensation)	Not visible
	Compensated (remapped to visible area)	Visible

As shown in Table 1, for all three types of simulated visual field defects, the test signage was not visible when the compensated visual information was projected onto the non-visible (defective) region. In contrast, when the information was remapped and projected onto the participant’s intact visible area, the signage became clearly perceivable. This pre- and post-comparison experimental design intuitively demonstrates the clinical effectiveness of the system. It also provides specific data for quantitative performance evaluation. By analyzing the test results obtained under different visual field defect patterns, we can further elucidate the system’s core role in personalized compensation. Specifically, these results verify that the system can dynamically adjust its visual enhancement strategies according to each participant’s unique pattern of visual field loss, effectively transforming previously inaccessible visual information into perceivable signals within the residual functional field of view. This approach maximally improves situational awareness and reduces the impact of visual field defects.

## 4 Discussion

We developed an AR-based visual field assistance system. It is designed for elderly people with low vision. This is because older adults are more prone to age-related vision deterioration, particularly in the presence of cumulative metabolic and environmental risk factors (Trujillo-Tanner et al., 2018; Trauzettel-Klosinski, 2018). Previous studies have mainly focused on magnification to improve vision in people with low vision (Hernandez-Moreno et al., 2023; Christen and Abegg, 2016). However, magnification technology cannot restore the vision of people with visual impairments (Miller et al., 2024). This leads to great dangers in daily life. Our instrument detects this risk factor and uses AR technology to expand the patient’s field of vision, achieving a dual improvement in vision and visual field (Zhao, 2023). This device is even more beneficial for their daily lives (Thomas et al., 2015). It should be noted that this device is not suitable for patients with extremely severe vision loss and almost no remaining vision. On the contrary, it is particularly suitable for patients who have focal visual field defects (such as hemianopia, quadrant blindness, tubular vision, etc.) while the remaining areas of the visual field are normal or only slightly impaired. Some studies have utilized smartphones and applications as auxiliary tools to provide magnification and zooming functions for people with low vision (Tan et al., 2024; Lee et al., 2020). It provided significant assistance to people with low vision. Our further efforts aim to expand the visual field for people with low vision. Furthermore, patients do not need to make complex cognitive judgments or operational decisions. They simply need to wear AR glasses normally, connect their smartphones, and complete basic operations by clicking the screen of the phone (Robinson et al., 2017). Then, they can perceive the compensatory image through natural vision. The goal is to help with their visual field problems. It also aims to improve their functional vision and overall quality of life. The system has two main parts. One part helps with clinical diagnosis of visual field issues. The other uses AR to expand the user’s visual field. Together, they form a personalized, multi-function visual aid.

Visual impairment can also affect spatial awareness. Due to the decline in depth perception and contrast sensitivity, the risk of accidents and falls increases (De Boer et al., 2004; Lord, 2006). Although there have been studies that have mentioned the application of AR technology to the understanding of visual impairments (Author anonymous, 2017; Loomis et al., 1993; Yu and Saniie, 2025). But our study introduces a new idea. It applies AR technology to build a complete low-vision rehabilitation system. The system combines three things: diagnostic help, visual field compensation, and safety alerts. Traditional low-vision aids often have only one function or limited technology. They cannot handle complex or changing visual needs (Musa et al., 2025). In contrast, our system uses AR to place virtual information onto the real world instantly. This makes visual field expansion and information display more intuitive. By bringing these different technologies together, we go beyond the limits of conventional devices. This creates a new direction for low-vision rehabilitation and has strong theoretical value.

The system also handles different types of visual field defects well. This is another important point. It works closely with clinical visual field diagnostic tools. From these tools, it gets accurate data about each patient’s defect. That includes the size, depth, and shape of the missing areas. Then the system changes the AR visual field expansion and safety alerts based on that personal data. As a result, each user gets a customized visual aid. This is a big change from the old “one-size-fits-all” method. Moving to a patient - specific approach makes the assistance more precise. It also offers useful ideas for future smart rehabilitation devices (Yang et al., 2026; Duan et al., 2026).

This study is a good example of interdisciplinary work. It brings together ophthalmology, biomedical engineering, and computer science. We take medical mechanisms, clinical diagnostic criteria, and the rehabilitation needs of elderly low-vision patients. Then we turn them into engineering requirements. We also use advanced AR technology. In this way, the research bridges the gap between medical theory and engineering practice. This interdisciplinary model helps promote the use of related technologies in medicine. It also offers a useful reference for future innovative research at the intersection of medicine and engineering.

We have made good progress, but there are still some limitations. Future work needs to address them.

Hardware performance and portability. Current AR glasses could be better in terms of size, weight, and comfort. If elderly users wear them for a long time, they may feel uncomfortable. This can reduce their willingness to use the system. Battery life is another issue. It limits how often the glasses can be used in daily life. Future research should focus on making the hardware smaller, lighter, and more power-efficient. That would improve the user experience.

User acceptance and adaptability. Using new technology takes some learning and getting used to. Some elderly users may have trouble with this. Their cognitive function may not be as good. Or they may simply not like new devices. For them, wearing AR glasses, using the system, and understanding the information on the screen could be hard. Future work should focus more on user interface and user experience design. We need to develop interaction methods that are simpler and more intuitive. It is also important to provide proper user training and to run systematic tests on how well users adapt to the system.

Long-term effects and safety. This study mainly focused on system design and basic functional testing. But there is more to do. For example, we need to see how much the system improves functional vision and quality of life over time. We also need to check if it causes any harm to eye health or creates psychological stress. To get reliable answers, we should run large-scale, multicenter, randomized controlled trials. At the same time, we must monitor engineering factors like reliability, stability, and failure rate during long-term use in real-world settings.

Experimental design and error analysis. Our validation was conducted on healthy participants with simulated defects rather than real patients. The current study did not systematically quantify false positives or false negatives. Based on our experimental observations, false negatives occasionally occurred when participants were distracted or when fixation drifted from the central cross during prolonged testing sessions, while false positives were rare. However, these observations remain qualitative, and systematic quantification of these error metrics is necessary to evaluate system reliability. Additionally, formal reliability testing (e.g., test–retest reliability, intraclass correlation coefficient) has not been performed, as the primary objective of this initial technical validation was to demonstrate feasibility and functional efficacy under controlled conditions. Future work will address these issues by recruiting a larger cohort to conduct test–retest reliability analyses on separate days, calculating intraclass correlation coefficients for both visual field mapping and compensation outcomes, quantifying false positive and false negative rates under standardized conditions, and assessing inter-rater reliability when operated by different technicians.

## 5 Conclusion

This study looks at a visual field assistance system. The system uses augmented reality and smartphones. It is designed for elderly people with low vision. This system first conducts a customized visual inspection test to identify the areas of visual impairment for the user. Subsequently, it uses an AR display equipped with a smartphone to re-map the external visual information onto the patient’s normal visual field area. The experimental results show that the compensation technology based on AR continuously enhances the stimulus detection ability in three types of simulated defects (cylindrical field of view, ipsilateral amblyopia, and central scotoma). After compensation, the detection changes from “invisible” to “visible”. These findings provide quantitative evidence that the system can effectively restore the perception of previously invisible environmental information. It also expands the functional field of view in a controlled environment. Despite these remaining steps, this study establishes a practical and evidence-based foundation for AR-assisted visual rehabilitation, and offers a promising complement to existing low-vision aids.

## Data Availability

The original contributions presented in the study are included in the article/supplementary material, further inquiries can be directed to the corresponding authors.

## References

[B1] Abraham C. H. Sakyi-Badu G. Boadi-Kusi S. B. Morny E. Darko-Takyi C. Ocansey S. (2024). Simulation of visual impairment in persons with normal vision for scientific research. Ophthalmic Physiol. Opt. 44 (2), 442–456. 10.1111/opo.13268 38223917

[B2] Agarwal R. Tripathi A. (2021). Current modalities for low vision rehabilitation. Cureus 13 (7), e16561. 10.7759/cureus.16561 PMC8396411 34466307

[B3] Ager L. (1998). Optical services for visually impaired children. Community Eye Health 11 (27), 38–40. 17492038 PMC1706058

[B5] Bjelica M. Levine I. C. Novak A. C. (2021). Increasing the contrast of tread edge highlighters improves stair descent safety in older adults with simulated visual impairment. Appl. Ergon. 97, 103525. 10.1016/j.apergo.2021.103525 34256320

[B7] Bourne R. R. A. Steinmetz J. D. Flaxman S. Briant P. S. Taylor H. R. Resnikoff S. (2021). Trends in prevalence of blindness and distance and near vision impairment over 30 years: an analysis for the global burden of disease study. Lancet Glob. Health 9, e130–e143. 10.1016/s2214-109x(20)30425-3 PMC7820390 33275950

[B8] Bruno R. R. Wolff G. Wernly B. Masyuk M. Piayda K. Leaver S. (2022). Virtual and augmented reality in critical care medicine: the patient's, clinician's, and researcher's perspective. Crit. Care 26 (1), 326. 10.1186/s13054-022-04202-x 36284350 PMC9593998

[B9] Cao D. Yao J. Ting D. Tan G. (2025). Artificial intelligence in predicting anti-VEGF treatment response in diabetic macular edema: current progress and future directions. Vis. Neurosci. 42, e027. 10.48130/vns-0025-0027

[B10] Cheng C. Y. Sheu M. M. Chen P. S. Chuang Y. T. Cheng H. M. Hsieh H. P. (2023). Assessing low vision care and the utilisation of optical low vision aids among patients with visual impairment in Taiwan. Ophthalmic Physiol. Opt. 43 (6), 1356–1363. 10.1111/opo.13205 37476932

[B11] Christen M. Abegg M. (2016). The effect of magnification and contrast on reading performance in different types of simulated low vision. J. Eye Mov. Res. 10 (2), 1–9. 10.16910/jemr.10.2.5 PMC7141081 33828652

[B12] Christensen K. Doblhammer G. Rau R. Vaupel J. W. (2009). Ageing populations: the challenges aheadLancet. 374, 1196–1208. 10.1016/S0140-6736(09)61460-4 19801098 PMC2810516

[B13] Dalal D. M. Shah D. (2022). Low vision in children: adaptation versus prescribing devices. Acta Sci. Ophthalmol. 5, 2582–3191. 10.31080/asop.2022.05.0576

[B6] De Boer M. R. Pluijm S. M. F. Lips P. Moll A. C. Völker-Dieben H. J. Deeg D. J. H. (2004). Different aspects of visual impairment as risk factors for falls and fractures in older men and women. J. Bone Min. Res. 19, 1539–1547. 10.1359/JBMR.040504 15312256

[B58] Delikaya M. Hamdi R. Bereuter C. Schroeter J. Oertel F. C. (2026). An update on the developments and challenges with the diagnosis and classification of autoimmune optic neuritis. Exp. Rev. Neurotherapeutics 26 (4), 381–398. 10.1080/14737175.2026.2632795 41688447

[B15] Duan N. Zhao X. Wu Z. Yuan D. Peng S. Cui K. (2026). Exploring the capabilities of three artificial intelligence chatbots in diagnosis and decision-making of age-related macular degeneration. Br. J. Ophthalmol. 10.1136/bjo-2025-328860 42270292

[B16] Edalati S. Slobin J. Harsinay A. Vasan V. Taha M. A. Del Signore A. (2024). Augmented and virtual reality applications in rhinology: a scoping review. Laryngoscope 134 (11), 4433–4440. 10.1002/lary.31602 38924127

[B17] Flaxman S. R. Bourne R. R. A. Resnikoff S. Ackland P. Braithwaite T. Cicinelli M. V. (2017). Global causes of blindness and distance vision impairment 1990–2020: a systematic review and meta-analysis. Lancet Glob. Health 5, e1221–e1234. 10.1016/S2214-109X(17)30393-5 29032195

[B18] Gagliardi O. M. Alisi L. Giacomo V. Albanese G. M. Scordari S. Marenco M. (2025). OCT predictors of retinal atrophy in neovascular age-related macular degeneration treated with aflibercept. Int. J. Ophthalmol. 18 (4), 648–655. 10.18240/ijo.2025.04.11 PMC11947533 40256033

[B19] Hernandez-Moreno L. Senra H. Lewis P. Moreno N. Linhares J. Santana R. (2020). Cost-effectiveness of basic vision rehabilitation (the basic VRS-effect study):study protocol for a randomised controlled trial. Ophthalmic Physiol. Opt. 403, 350–364. 10.1111/opo.12665 31989690

[B20] Hernandez-Moreno L. Senra H. Marques A. P. Perdomo N. M. Macedo A. F. (2023). The basic VRS-effect study:clinical trial outcomes and cost-effectiveness of low vision rehabilitation in Portugal. Ophthalmol. Ther. 121, 307–323. 10.1007/s40123-022-00600-0 36369618 PMC9651903

[B21] Jackson M. L. Schoessow K. A. Selivanova A. Wallis J. (2017). Adding access to a video magnifier to standard vision rehabilitation: initial results on reading performance and well-being from a prospective, randomized study. Digit. J. Ophthalmol. 23 (1), 1–10. 10.5693/djo.01.2017.02.001 28924412 PMC5596951

[B22] Wei J. W. Zhang Q. Wang X. Y. Huang M. X. Gan J. H. Yang W. H. (2026). Advances in the application of virtual reality technology in ophthalmic surgical skills training. Int. J. Ophthalmol. (2), 393–404. 10.18240/ijo.2026.02.23 41572988 PMC12820628

[B23] Kaur K. Gurnani B. (2023). “Low vision aids,” in StatPearls. (Treasure Island, FL: StatPearls Publishing). 36256771

[B24] Lee H. N. Ashok V. Ramakrishnan I. V. (2020). Screen magnification for office applications. ASSETS. 2020 95. 10.1145/3373625.3418049 PMC7871702 33569549

[B25] Liu L. Xu L. Yu B. Zhao L. Wu H. (2024). The influence of simulated visual impairment on distance stereopsis. J. Vis. 24 (2), 1. 10.1167/jov.24.2.1 38300556 PMC10846341

[B26] Loomis J. Golledge R. Klatzky R. (1993). “Personal guidance system for the visually impaired using GPS, GIS, and VR technologies,” in Proc. Conf. on Virtual Reality and Persons with Disabilities, 17–18.

[B27] Loomis J. M. Klatzky R. L. Giudice N. A. (2018). “Sensory substitution of vision: importance of perceptual and cognitive processing,” in Assistive Technology for Blindness and Low Vision (Boca Raton, FL, USA: CRC Press), 179–210.

[B28] Lord S. R. (2006). Visual risk factors for falls in older people. Proc. Age Ageing 35, ii42–ii45. 10.1093/ageing/afl085 16926203

[B29] Luo X. Ye S. Sun B. Liu Y. Hu X. Wang L. (2026). Joint effects of visual acuity impairment and visual field loss on reading performance: a low-vision simulation in Chinese readers. Eye Vis. (Lond) 13 (1), 25. 10.1186/s40662-026-00497-x 42277949 PMC13262081

[B30] Manouchehri V. Albonico A. Hemström J. Djouab S. Kim H. Barton J. J. S. (2022). The impact of simulated hemianopia on visual search for faces, words, and cars. Exp. Brain Res. 240 (11), 2835–2846. 10.1007/s00221-022-06457-w 36069920

[B31] Miller A. Macnaughton J. Crossland M. D. Latham K. (2024). “I'm like something out of star wars”: A qualitative investigation of the views of people with age-related macular degeneration regarding wearable electronic vision enhancement systems. Disabil. Rehabil. 46 (19), 4476–4485. 10.1080/09638288.2023.2278179 37933205

[B32] Minto H. Butt I. A. (2004). Low vision devices and training. Community Eye Health 17 (49), 6–7. 17491789 PMC1705708

[B33] Musa M. Enaholo E. Bale B. I. D'Esposito F. Gagliano C. Giglio R. (2025). A review of optical and digital aids for magnification techniques in low-vision rehabilitation. J. Biol. Methods 12 (4), e99010072. 10.14440/jbm.0002 41415878 PMC12709523

[B35] Perrault M. A. Lauer G. Voss S. Seitz B. Käsmann-Kellner B. (2023). Visual impairment and low vision Aids-A comparison between children and adults. J. Pers. Med. 13 (11), 1608. 10.3390/jpm13111608 38003923 PMC10672320

[B36] Prunoiu V. M. Popa D. Şerbulea M. S. Brătucu E. Simion L. Brătucu M. N. (2022). Augmented reality in surgical oncology. A literature review. Chir. (Bucur) 117 (5), 517–525. 10.21614/chirurgia.2785 36318681

[B37] Reeves J. L. (2011). Vision should not be overlooked as an important sensory modality for finding host plants. Environ. Entomol. 40, 855–863. 10.1603/EN10212 22251686

[B38] Riazi A. Gharebaghi R. Heidary F. (2022). Seven-year experience in a low vision rehabilitation clinic at a tertiary referral center. Med. Hypothesis Discov. Innov. Optom. 3, 128–135. 10.51329/mehdioptometry161

[B39] Robinson J. L. Braimah Avery V. Chun R. Pusateri G. Jay W. M. (2017). Usage of accessibility options for the iPhone and iPad in a visually impaired population. Semin. Ophthalmol. 322, 163–171. 10.3109/08820538.2015.1045151 26154395

[B40] Runswick O. R. Rawlinson A. Allen P. M. Sharpe B. T. Pocock C. Datson N. (2023). The effects of simulated vision impairment on performance in football. J. Sports Sci. 41 (14), 1410–1422. 10.1080/02640414.2023.2273093 37877884

[B41] Sapkota K. Kim D. H. (2017). Causes of low vision and major low-vision devices prescribed in the low-vision clinic of Nepal eye hospital, Nepal. Anim. Cells Syst. Seoul. 21 (3), 147–151. 10.1080/19768354.2017.1333040 30460063 PMC6138330

[B43] Su S. Wang R. Chen Z. Zhou F. Zhang Y. (2023). Augmented reality-assisted versus conventional total hip arthroplasty: a systematic review and meta-analysis. J. Orthop. Surg. Res. 18 (1), 920. 10.1186/s13018-023-04421-0 38042852 PMC10693088

[B44] Tan H. L. Aplin T. McAuliffe T. Gullo H. (2024). An exploration of smartphone use by, and support for people with vision impairment:a scoping review. Disabil. Rehabil. Assist. Technol. 192, 407–432. 10.1080/17483107.2022.2092223 35776428

[B45] Thomas R. Barker L. Rubin G. Dahlmann-Noor A. (2015). Assistive technology for children and young people with low vision. Cochrane Database Syst. Rev. 2015 (6), CD011350. 10.1002/14651858.CD011350.pub2 26086876 PMC10638521

[B46] Trauzettel-Klosinski S. (2018). Current possibilities of visual rehabilitation. Ophthalmologe 11510, 895–910. 10.1007/s00347-018-0767-0 30128593

[B47] Trujillo-Tanner C. Caserta M. S. Kleinschmidt J. J. Clayton M. S. Bernstein P. S. Guo J. W. (2018). Conducting research with older adults with vision impairment:lessons learned and recommended best practices. Gerontol. Geriatr. Med. 4, 2333721418812624. 10.1177/2333721418812624 30515451 PMC6262493

[B48] United Nations Department Department of Economic and Social Affairs Population Division (2019). World population prospects 2019: highlights (ST/ESA/SER.A/423). Available online at: https://population.un.org/wpp/Publications/Files/WPP2019_Highlights.pdf (Accessed July, 292023).

[B49] Uno T. Matsuo T. Asano M. Loh P. Y. (2023). Effects of simulated visual impairment conditions on movement and anxiety during gap crossing. Healthc. (Basel) 12 (1), 42. 10.3390/healthcare12010042 PMC10779388 38200948

[B50] Vashist P. Senjam S. S. Gupta V. Gupta N. Kumar A. (2017). Definition of blindness under national programme for control of blindness: do we need to revise it? Indian J. Ophthalmol. 65 (2), 92–96. 10.4103/ijo.IJO_869_16 28345562 PMC5381306

[B34] van Nispen R. M. Virgili G. Hoeben M. Langelaan M. Klevering J. Keunen J. E. (2020). Low vision rehabilitation for better quality of life in visually impaired adults. Cochrane Database Syst. Rev. 1 (1), CD006543. 10.1002/14651858.CD006543.pub2 31985055 PMC6984642

[B14] van Doormaal J. A. M. van Doormaal T. P. C. (2024). Augmented reality in neurosurgery. Adv. Exp. Med. Biol. 1462, 351–374. 10.1007/978-3-031-64892-2_21 39523276

[B4] Williams F. (2017). OxSight uses augmented reality to aid the visually impaired. TechCrunch. Available online at: https://techcrunch.com/2017/02/16/oxsight-uses-augmented-reality-to-aide-the-visually-impaired/.

[B51] Wood J. M. (2022). Vision impairment and On-Road driving. Annu. Rev. Vis. Sci. 8, 195–216. 10.1146/annurev-vision-100820-085030 36108105

[B52] Yang W. Zheng Y. Wang S. (2026). Editorial: imaging in the diagnosis and treatment of eye diseases. Front. Ophthalmol. 6, 1899308. 10.3389/fopht.2026.1899308 PMC13322848 42395831

[B53] Yu X. Saniie J. (2025). Visual impairment spatial awareness system for indoor navigation and daily activities. J. Imaging 11 (1), 9. 10.3390/jimaging11010009 39852322 PMC11766877

[B54] Yuan L. Y. Li L. P. Hua X. Yuan X. Y. (2025). Tracing global progress: two decades of age-related macular degeneration research. Int. J. Ophthalmol. 18 (5), 925–936. 10.18240/ijo.2025.05.20 40385108 PMC12043290

[B55] Yuan L. Kang D. Dong X. Liu L. Grzybowski A. Jin K. (2025). Artificial intelligence in clinical education in ophthalmology: a systematic review. Vis. Neurosci. 42, e026. 10.48130/vns-0025-0025

[B56] Zhao Y. (2023). Invited session IV:Extended reality--applications in vision science and beyond:augmented reality systems for people with low vision. J. Vis. 2315, 18. 10.1167/jov.23.15.18

[B57] Zhao Z. Poyhonen J. Chen C. X. Sophie Woodley Hooper F. Ma Y. Hu Y. (2021). Augmented reality technology in image-guided therapy: state-Of-The-Art review. Proc. Inst. Mech. Eng. H. 235 (12), 1386–1398. 10.1177/09544119211034357 34304631 PMC8573682

